# Clustering of risk factors for non-communicable disease and healthcare expenditure in employees with private health insurance presenting for health risk appraisal: a cross-sectional study

**DOI:** 10.1186/1471-2458-13-1213

**Published:** 2013-12-21

**Authors:** Tracy L Kolbe-Alexander, Jaco Conradie, Estelle V Lambert

**Affiliations:** 1 Department of Human Biology, UCT Faculty of Health Sciences, UCT/MRC Research Unit for Exercise Science and Sports Medicine, University of Cape Town, P.O. Box 115, Newlands, Cape Town 7725, South Africa; 2 Discovery Health, Johannesburg, South Africa

**Keywords:** Clustering of risk factors, Physical activity, Healthcare expenditure

## Abstract

**Background:**

The global increase in the prevalence of NCD’s is accompanied by an increase in risk factors for these diseases such as insufficient physical activity and poor nutritional habits. The main aims of this research study were to determine the extent to which insufficient physical activity (PA) clustered with other risk factors for non-communicable disease (NCD) in employed persons undergoing health risk assessment, and whether these risk factors were associated with higher healthcare costs.

**Methods:**

Employees from 68 companies voluntarily participated in worksite wellness days, that included an assessment of self-reported health behaviors and clinical measures, such as: blood pressure (BP), Body Mass Index (BMI), as well as total cholesterol concentrations from capillary blood samples. A risk-related age, ‘Vitality Risk Age’ was calculated for each participant using an algorithm that incorporated multiplicative pooled relative risks for all cause mortality associated with smoking, PA, fruit and vegetable intake, BMI, BP and cholesterol concentration. Healthcare cost data were obtained for employees (n = 2 789).

**Results:**

Participants were 36 ± 10 years old and the most prevalent risk factors were insufficient PA (67%) and BMI ≥ 25 (62%). Employees who were insufficiently active also had a greater number of other NCD risk factors, compared to those meeting PA recommendations (chi^2^ = 43.55; p < 0.0001). Moreover, employees meeting PA guidelines had significantly fewer visits to their family doctor (GP) (2.5 versus 3.11; p < 0.001) than those who were insufficiently PA, which was associated with an average cost saving of ZAR100 per year (p < 0.01). Furthermore, for every additional year that the ‘Vitality Risk Age’ was greater than chronological age, there was a 3% increased likelihood of at least one additional visit to the doctor (OR = 1.03; 95% CI = 1.01 – 1.05).

**Conclusion:**

Physical inactivity was associated with clustering of risk factors for NCD in SA employees. Employees with lower BMI, better self-reported health status and readiness to change were more likely to meet the PA guidelines. These employees might therefore benefit from physical activity intervention programs that could result in improved risk profile and reduced healthcare expenditure.

## Background

It has been well established that the number of deaths attributable to non-communicable diseases (NCD’s) such as coronary artery disease, diabetes and hypertension are increasing globally [1]. This trend is evident in South Africa, where more than a third (37%) of all deaths are due to NCD’s [2]. The increase in the prevalence of NCD’s is accompanied by an increase in risk factors for these diseases such as insufficient physical activity, smoking and poor nutritional habits [3,4]. Furthermore, the burden of disease due to NCD risk factors was higher in 2010 than in 1990, and physical activity together with poor dietary habits accounted for 10% of global Disability life years (DALYS) in 2010 [5]. Low fruit and vegetable intake and physical inactivity each accounted for 1.% of total DALYs and were ranked as the 11th and 12th leading risk factors, respectively, in South Africa [6].

Physical inactivity appears to cluster with other risk factors for cardiovascular disease where those who are insufficiently physically active were more like to have additional risk factors such as elevated serum triglycerides, hypertension and elevate fasting glucose levels [7]. Similarly, the results of a study based on data from the United States National Health and Nutrition Examination Survey, shows that individuals who are inactive are more likely to have additional risk factors for cardio metabolic disease [8]. These findings are corroborated by a study conducted in Swedish men and women where those who had higher levels of physical activity had significantly lower triglycerides and less atherogenic lipid profiles compared to those who were inactive [9]. Furthermore, the Swedes who had higher fitness levels were 50% less likely to have additional three or more risk factors for NCD [9].

The worksite has been shown to be a favorable setting to implement intervention programs aiming to reduce the risk for and prevalence of NCD’s, as many individuals can be reached at the same time [10]. These programs have been shown to play a role in improving health status and lifestyle behaviors, such as increased physical activity and reduced dietary fat intake among employees [11]. The Health Risk Assessment (HRA) often represents the first step in preventive screening and an entry point for employee wellness programs [12]. The components of the HRA may vary, but often includes a medical history, self-reported health status, and lifestyle behaviors, as well as readiness to change for specific lifestyle behaviors [13].

The implementation of an HRA at a worksite is largely dependent on individuals volunteering their participation and the aggregated results may therefore not be generalizable to the worksite, as a whole, or more broadly, to the population of employed persons. Indeed, previous research has shown that employees completing HRA’s are usually older, report fewer days of sick leave and have better self-reported health status, than those who do not [13,14]. However, describing the clustering of risk behavior amongst participants may provide further insight into the characteristics of individuals who choose to participate in these types of health promotion activities, and also facilitate effective strategies to recruit new participants. Thus the messaging and advertising encouraging HRA participation could be directed at encouraging younger and less healthy employees to participate. In addition, some employees have listed lack of time as a barrier for participation, thus future strategies might include having shorter HRA’s in order to encourage higher rates of participation [14].

HRA results have also been associated with prospective medical expenses [11]. For example, Pronk and colleagues found that healthcare costs in the year following a HRA were lower for employees with healthier lifestyle behaviors and better health status [11]. These authors calculated a number of scores which included the “modifiable potential health score” (MPHS), comprised of physical activity behavior, tobacco use, diet quality, breakfast consumption, fruit and vegetable consumption, calcium, sugar intake, sleep, alcohol use and self reported stress [11]. A higher MPHS was significantly associated with future annual healthcare costs, *F*(46) = 26.43; p < 0.001 [15].

Consequently, some private health insurance companies offer wellness days and opportunities for employees to complete a HRA in order to determine their current health status. Private health insurance coverage is relatively low in South Africa where only 16% of South Africans have private health insurance [16,17]. Being employed has been identified as one of the main predictors of having private healthcare insurance [18], which might be largely due to some worksites including compulsory private healthcare cover as a condition of service [19].

There are limited data from South Africa exploring the association between physical activity behavior and additional risk factors for non-communicable disease (NCD) and healthcare expenditure among employees who have private health insurance. Therefore, the main aim of this study was to determine the extent to which insufficient physical activity clustered with other risk factors for NCD in employed persons presenting for health risk assessment as part of worksite wellness day. A second aim was to determine whether there was an association between increased number of risk factors for NCD and healthcare expenditure. Thirdly, we aimed to compare healthcare expenditure for employees participating in the worksite wellness day with those who did not participate.

## Methods

This is a cross-sectional research study using secondary data analysis for employees based at worksites who hosted a health and wellness day for their staff.

### Setting

South Africa’s largest private health insurer offers their corporate clients an opportunity to host one wellness day for their employees annually, during which health screening and self-reported behaviors are assessed. Accredited service providers who conduct all the measurements at the wellness days are required to comply to the health insurer’s standard measurement guide and calibration of equipment in order to ensure that all measures are accurate and reliable. The cost of the wellness day is shared between the private health insurer and the employer, thus the employee does not make any financial contribution towards the services.

All employees from these worksites were invited to participate in a one-day health and wellness event. They participated on a voluntary basis, and all information gathered remained confidential and was not made available to management or human resource departments. Each worksite only has one wellness day per year therefore the employees only have one opportunity to participate per calendar year. This analysis comprises an evaluation of data that were collected over a 12-month period (January to December), from 68 companies. These worksites included companies from various sectors including engineering, logistics, consultants, information technology, academic, financial and transport sectors.

In all instances, the wellness days were conducted during normal work hours. There were no exclusion criteria, with the only prerequisite for participation being that the individual was an employee of the respective company. Employees’ data were recorded using unique identity codes, allocated by the private health insurance administrators, ensuring anonymity to researchers for secondary analysis.

The health insurance company forwarded the unlinked data (no personal identifiers; all data coded) results of the health risk assessment and clinical measures to the researchers for data analysis. Ethical approval for this research study was obtained from the University of Cape Town’s Research and Ethics Committee of the Faculty of Health Sciences (REF 348/2008).

### Participants

**Figure 1:**
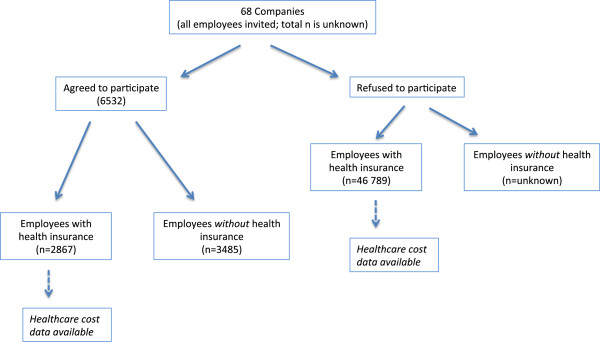
Description of participants and non participants for the health insurer’s health and wellness day.

Employees (n = 6 532) from worksites (n = 68) conducting wellness days participated on a voluntary basis. However, the data analysis only included those employees who were clients of the private health insurer (n = 2 867), as the healthcare expenditure data was available for this group only (Figure 1).

In addition to employees who participated in the wellness screening, the private health insurer provided health-care expenditure for employees who did not attend the screening (non attenders, n = 46 789) but who were also their clients. Thus, the non-participants were selected on the basis of incidence sampling, and represent the employees who did not participate in the annual wellness days. These data were sent without any personal identifiers, and allowed the researchers to compare the healthcare expenditure of employees who attended the health screening to those who did not participate.

### Measurements

#### Health Risk Assessment (HRA) questionnaire

The HRA included questions on participant demographics (age, gender), medical and family history, self-reported modifiable lifestyle behaviors and intention to change or improve these behaviors. Participants reported the number physical activity sessions in a usual week, the duration and intensity of each session, from which the average minutes of at least moderate physical activity per week was calculated. The average number of daily servings of fruit and vegetables were recorded. In addition, employees reported on whether they were never smokers, ex-smokers or current smokers.

#### Clinical measures

Trained staff, including Exercise Physiologists and nurses, performed all clinical measurements on the wellness days. These measurements included screening tests for cholesterol concentrations*,* using finger-prick capillary blood samples (Accutrend® GC analysers, Roche Diagnostics). Blood pressure was measured twice per person using an automated sphygmomanometer and the average of the two readings was recorded. Employees sat quietly for approximately five minutes before being measured.

Standing *height* (cm) was measured to the nearest centimeter, using a stadiometer and *body weight* was measured using a portable calibrated scale and recorded to the nearest 0.1 kg. Body Mass Index (BMI) was calculated as body mass (kg) divided by height (m) squared (kg/m^2^).

#### Total risk score

A total risk score was calculated for each participant based on seven risk factors. A score of one was allocated if an employee was above the threshold for a specific risk factor. If the employee was below the threshold for the risk factor, they were given a score of zero. The risk factors and threshold included in the calculation for total risk included age (≥ 45 years for men and ≥ 55 years for women), having a Body Mass Index (BMI) more than 24.9 kg/m^2^[20], blood pressure greater than 140/90 mmHg [21], and a total cholesterol reading greater than 5.2 mmol/l [22]. Lifestyle behaviors for risk classification included consuming less than five servings of fruit and vegetables [23] per day and being a smoker [24]. Those employees who participated in less than 150 minutes of at least moderate physical activity per week were also categorized as being at risk for NCD [25]. Thus the maximum total risk score was seven and the theoretical minimum score was zero.

Employees were further subdivided according to the total number of risk factors for CVD based on the American College of Sports Medicine (ACSM) criteria. ASCM classifies individuals with two or more risk factors as being at moderate risk for CVD and those with one or less risk factors being low risk for CVD.

#### Risk-related age

A risk-related age, modified from ‘Vitality Risk Age’ was calculated for each participant using an algorithm that incorporated multiplicative pooled relative risks for all cause mortality associated with smoking, physical activity, fruit and vegetable intake, BMI and cholesterol concentration [26]. ‘Vitality Risk Age’ was purpose-built for creating awareness and “health messaging” and to encourage lifestyle behavior change and to be used for risk stratification and risk management at a corporate level. The variables that lead to a higher ‘Vitality Risk Age’ included: increased BMI, elevated serum cholesterol concentrations, elevated blood pressure, diabetes and being a current smoker [26]. Meeting physical activity and fruit and vegetable guidelines resulted in a lower ‘Vitality Risk Age’. The difference between chronological age and the ‘Vitality Risk Age’ was calculated, as an indicator of total risk for all-cause mortality.

#### Healthcare expenditure

Healthcare cost data, quantified in South African Rands, ZAR (1 US $ ≈ 8.8 ZAR), were obtained and included the number of visits and associated costs to the hospital and the family doctor (General Practitioner/GP). Expenditure related to chronic disease medication and total healthcare expenditure was also obtained. These data were only available for employees who were clients of the private health insurer.

### Statistical analysis

STATISTICA software package was used for all the analyses (Stasoft, Inc. 184–199, Tulsa OK, USA). Descriptive statistics were performed for the total sample and included calculating the mean, standard deviation and standard error for all continuous variables. Because physical activity data were not normally distributed, the median and quartile values were presented for minimum and maximum weekly physical activity. Frequency tables were used to determine the percentage of individuals at risk, and also for the stages of change data. We conducted a one-way ANOVA to determine whether there were any significant differences in healthcare expenditure among employees who participated in the wellness days and those who did not participate. In addition, the One-Way ANOVA was computed to determine if there were any significant differences between the men and women who participated in the wellness day.

The Chi^2^ analysis was performed to determine whether those employees who met physical activity guidelines had fewer additional cardiovascular disease risk factors than those who were insufficiently physically active. An analysis of variance (ANOVA) was used to determine if there were differences in healthcare expenditure between persons meeting physical activity guidelines versus those who were insufficiently active. Similarly, participants were grouped into those with more than 2 risk factors for CVD and those with less than two risk factors, and an ANOVA was performed to determine whether there were significant differences in healthcare expenditure between these two groups. Age and gender were used as covariates in both ANOVA models.

Multiple linear regression analysis was conducted to examine the factors associated with meeting physical activity guidelines. The variables included in this model were age, use of chronic medication and number of total number of modifiable risk factors (excluding physical activity) for NCD. Likewise, a multiple regression analysis for doctors visits was performed which included age, use of chronic medication and modifiable risk factors (excluding physical activity) and habitual levels of physical activity.

Finally logistic regression models were computed to determine the odds of being classified as ‘at risk’ for each of the other NCD risk factors in addition to being insufficiently active. Additional logistic regression analyses were performed to determine the odds of hospitalization and an additional visit to the doctor based on each year that the ‘Vitality Risk Age’ is more than chronological age.

## Results

### Wellness day participants versus non-participants

Table 1 provides a comparison of healthcare costs for wellness day participants (n = 2 867) and non-participants (n = 46 789). The total number of non-participants in the analysis included only those employees who were clients of the private health insurer. The non-participants were comprised of those who chose not to attend the wellness days, who were not at work due to illness, or those who were unable to attend due to logistical constraints, such as being off-site or on another shift. We were unable to separate the non-participants into these sub-groups.

**Table 1:** Comparison of healthcare costs between wellness day participants and employees who did not participate

	**Participated in wellness day**	**Did not participate in wellness day**	**P-value**
	**(n = 2867)**	**(n = 46789)**	
**GP Visits** (number in past 12 months)	2.86; 2.92-3.07	2.96; 3.07-3.11	>0.05
**Hospital Admissions** (number in past 12 months)	0.2; 0.53-0.56	0.31; 0.79-0.80	<0.0001
**Chronic Medication** (cost in ZAR)	466; 4 132–4 345	1 313; 4 579–4 819	<0.0001
**GP Visits** (cost in ZAR)	571; 736 - 774	644; 909 - 921	<0.0001
**Hospital Admissions** (cost in ZAR)	2 773;12 633–13 284	6 104;27 476–27 830	<0.0001
**Total healthcare expenditure** (cost in ZAR)	7 277;16 357 – 17 200	12 341; 33 305–33 735	<0.0001

(Mean; 95% CI).

Legend: ZAR – South African Rands.

Wellness day participants had significantly lower chronic medication-related costs than the non-participants (Table 1). Similarly, the participants had significantly fewer hospital admissions and related costs. Despite no difference in the number of visits to the primary healthcare doctor, the General Practitioner (GP), the non-participants had significantly higher GP related expenditure than the participants, as represented in Table 1. Furthermore, the total healthcare expenditure was significantly higher amongst the non-participants (mean ZAR 12 341, 95% CI 33 305–33 735) than the wellness day participants (mean ZAR 7 277, 95% CI 16 357–17 129) (Table 1).

#### Participant characteristics

The mean age for men and women was not statistically different (Table 2). The mean Body Mass Index (BMI) was more than 25 for both men and women, which is in the overweight category. The men reported consuming significantly fewer servings of fruit and vegetables per day and were significantly more physically active than the women. The women had significantly lower systolic blood pressure and fewer risk factors for coronary artery disease than the men (Table 2).

**Table 2:** Health and lifestyle characteristics of wellness day participants (mean ± SD)

	**Total (2 867)**	**Men (n = 635)**	**Women (n = 729)**	**P-value**
**Age** (years)	35.9 ± 10.1	35.6 ± 10.2	35.5 ± 9.7	NS
**BMI** (kg/h^2^)	26.6 ± 5.4	26.6 ± 4.4	26.4 ± 6.1	NS
**Cholesterol** (mmol/l)	4.8 ± 1.0	4.8 ± 1.1	4.8 ± 1.0	NS
**Systolic BP** (mm Hg)	123.8 ± 15.9	129.7 ± 18.8	120.0 ± 13.7	<0.0001
**Diastolic BP** (mm Hg)	79.7 ± 11.5	83.0 ± 11.1	77.4 ± 10.9	NS
**Fruit and Vege** (servings/day)	2.8 ± 1.6	2.6 ± 1.6	2.8 ± 1.7	0.042
**Physical activity** (min/wk)	^#^126.5 (75.0)	^#^144.6 (112.5)	^#^109.9 (56.25)	<0.0001
**Total risk factors** (number)		2.8 ± 1.2	2.6 ± 1.14	<0.0001

BMI: Body Mass Index; BP: blood pressure; vege: vegetables; Physical Activity: average minutes physical activity per week (self reported); ^#^indicates median values reported for average weekly physical activity; Total Risk factors = total number of risk factors for non-communicable diseases; NS = no significant differences between men and women, p > 0.05.

More than two thirds of employees (67%) did not meet the recommended 150 minutes of at least moderate physical activity per week. Sixty-two percent of employees were either overweight or obese and 71% consumed less than five servings of fruit and vegetables per day. In addition, 62% were either non-smokers or ex-smokers. Finally, one third of the employees had blood pressure more than 140/90 mmHg, and 41% had cholesterol values more than 5.2 mmol/l for the finger prick test.

The total number of risk factors was calculated for each participant. Only 17% of employees had fewer than 2 risk factors and the most prevalent number of risk factors was 3, which was found in 32% of the employees.

### Clustering of risk factors with physical activity

Table 3 compares the additional number of risk factors for those who were classified as being insufficiently physically active compared to those who were meeting physical activity recommendations. Those employees who were meeting the physical activity guidelines had significantly lower than expected occurrence of other modifiable risk factors for NCD than those who were categorized as being insufficiently active (Chi^2^ = 43.55 and p < 0.0001). These results were confirmed with the Kolmogorov-Smirnov test which showed that those who were sufficiently active had significantly fewer risk factors for coronary artery disease than those who were insufficiently physically active, 1.67 ± 1.1 versus 1.96 ± 1.1, p < 0.05.

**Table 3:** Physical activity risk and number of employees with additional risk factors for non-communicable disease

	**0 additional risk**	**1 additional risk**	**2 additional risk**	**3 additional risk**	**4 additional risk**
**Physical Activity Risk**					
**No**	88	223	211	114	30
**Yes**	168	591	815	481	157

Furthermore, those employees who were classified as being insufficiently active were 27% more likely to be overweight or obese (OR = 1.27; 95% CI = 1.15; 1.39) (Table 4). Similarly the odds of having elevated total cholesterol concentration and increased blood pressure increased by 17% and 23%, respectively for those who were inactive (OR = 1.17; 95% CI = 1.00; 1.37 and OR = 1.23; 95% CI = 1.05; 1.45). Physical activity risk was not significantly associated with increased odds of being a smoker.

**Table 4:** The increased odds of having additional risk factors for NCD [OR (95% confidence interval)], p-value

	**Physically active**	**Insufficiently active**	**p-value**
**BMI ≥ 25** (kg/h^2^)	Ref: 1.00	1.27 (1.15;1.39)	<0.01
**Cholesterol > 5.2** (mmol/l)	Ref: 1.00	1.17 (1.00; 1.37)	0.04
**Systolic BP > 140** (mm Hg)	Ref: 1.00	1.20 (1.00; 1.45)	0.04
**Diastolic BP >90** (mm Hg)	Ref: 1.00	1.30 (1.10; 1.55)	<0.01
**Fruit and Vegetables** (<5 servings per day)	Ref: 1.00	2.04 (1.74; 2.41)	<0.01
**Current smoker**	Ref: 1.00	1.01 (0.96; 1.08)	0.64

BMI: Body Mass Index; BP: blood pressure.

#### Healthcare expenditure

The multiple regression model which included age and use of chronic medication, showed that meeting physical activity guidelines had a marginal but significant association with the number of visits to the doctor (r^2 =^ 0.04; p < 0.001). Those who were insufficiently active had significantly higher number of visits than those who were meeting physical activity guidelines, 2.91 (95% CI: 2.8; 3.0) and 2.67 (95% CI: 2.44; 2.88), respectively, p = 0.04. Similarly, employees with two or more risk factors had significantly higher numbers of visits to the doctor in a 12-month period, and this was coupled with significantly higher health-care expenditure (related to doctors’ visits), after adjusting for age and gender (Table 5). There were no significant differences in healthcare expenditure related to chronic medication, hospital admissions and total healthcare expenditure for those who were and those who were not meeting physical activity recommendations (data not shown).

**Table 5:** Doctors visits and healthcare expenditure based on number of NCD risk factors (mean; 95% CI)

	**<2 risk factors**	**≥ 2 risk factors**	**p-value**
**Doctors visits** (number in previous 12 months)	2.5 (2.33; 2.66)	3.11 (2.96; 3.26)	<0.001
**Healthcare Expenditure ZAR** (related to Dr’s visits)	502.2 (465.6; 538.8)	616.6 (577.3; 655.9)	<0.001

Legend: ZAR – South African Rands.

<2 represents those with less than two risk factors for non-communicable disease.

≥2 represents having two or more risk factors for non-communicable disease.

Furthermore, for every additional year greater difference between the Vitality Risk Age and chronological age, there was a 3% increased likelihood of at least one additional visit to the doctor (OR = 1.03; 95% CI = 1.01 – 1.05). Similarly, the odds of hospital admissions increased by 4% for each year that the Vitality Age was higher than chronological age (OR = 1.04; 95% CI = 1.02 – 1.06).

## Discussion

The first finding in the present study was that the employees attending the wellness day screening event, which included completing a HRA, had significantly lower healthcare expenditure than those who did not attend. This finding is supported by other studies that have reported that employees attending wellness days or completing HRA’s have lower healthcare expenditure [24], are usually older, report fewer days of sick leave and have better self-reported health status, than those who do not [13]. For example, a recent study among Dutch employees found that those who completed the HRA had fewer sick leave days in the previous year than the non-participants [14]. Furthermore, significantly more Dutch employees who completed a HRA reported that their current health status was excellent than the non-participants [14]. Therefore, these results suggest that the employees who participated in the current study represent the ‘worried well’ [14]. Consequently, the health profile of all employees may actually be worse than that reported in our results.

One of our main findings was that those employees who were insufficiently physically active had a higher number of additional modifiable risk factors for NCD’s than those who were meeting physical activity recommendations. This suggests a clustering of risk factors with insufficient physical activity. These results are supported by those of a recent a cross-sectional study among Swedish men and women [9]. The odds of the physically active Swedish participants having three or more risk factors for cardiovascular disease were 50% lower than those who were inactive, even after adjusting for confounders [9]. Furthermore, in the Swedish study, the odds of having additional risk factors, such as hypertension or overweight, were further reduced among the participants with higher levels of cardiovascular fitness [9].

Conversely, Shi et al., [27] did not report an association between physical activity and additional risk factors for cardio-metabolic disease [27]. The effect of a combination of lifestyle behaviors, including dietary habits, smoking status, alcohol intake and physical activity, on cardio-metabolic risk was investigated in participants in the Lipid Research Clinic’s Princeton Follow-up study [27]. Cardio-metabolic risk was defined as having three or more clinical measures above recommended cut-points [27]. The participants that accumulated more than 4 hours of physical activity per week were less likely to have three or more additional risk factors for cardio-metabolic disease, but this association was not statistically significant [27]. Similarly, those who watched less than two hours of television per day were less likely to have additional risk factors for cardio metabolic disease (not statistically significant) [27]. The difference in results between this study and the current study are likely due to a more rigid threshold used for the physical activity threshold for risk, and defining ‘at risk’ as the presence of 3 or more risk factors. The ‘Lipid Research Clinic’s Princeton Follow-up study’, investigated the relationship between more than 4 hours of physical activity per week, whereas our research study used the cut point of 2.5 hours per week. It is worth noting that the current recommendation for physical activity is 2.5 hours per week, thus the cut point of 4 hours per week in the Lipid Research Clinic’s study may have reduced the sensitivity for finding an association.

The association between physical activity and reduced burden of disease may be mediated by intermediates in the causal pathway. For example, Rennie et al., found that physical activity reduced the likelihood of metabolic syndrome for both men and women [28]. Moderate intensity physical activity has been inversely correlated to waist to hip ratio in women, and BMI, as well as total cholesterol and trigyceride concentrations in men [28]. Similarly, men and women participating in vigorous intensity physical activity were less likely to have metabolic syndrome, OR = 0.50; 95% CI 0.39, 0.64, even after adjusting for age and other NCD risk factors [28]. These findings are supported by Pronk and Kottke who reported that adults who are physically active have a more favourable bio-maker profile and lower rates of all cause mortality than those who are inactive [15]. Furthermore, a recent research study among university employees reported that those who were inactive reported greater interest in health promotion programs [29]. Thus both completing the HRA and the associated results might be a valuable teachable moment to improve lifestyle behaviors and health status. Therefore, interventions that aim to increase habitual levels of physical activity could result in reducing concomitant risk factors and improve overall health status.

Another important finding from the current study was that those participants with 2 or more risk factors had significantly more visits to the doctor, and subsequently, higher associated healthcare expenditure. Those persons whose overall risk, based on modifiable health risks, were increased had overall greater health care utilization. From a practical and application point of view, these differences in costs are relatively small, despite being clinically significant. However, the potential total cost savings that can be accrued if a number of employees are able to have lower healthcare claims is large and might therefore be of both statistical and practical significance.

Since nearly three-quarters (71%) of the participants in our study were insufficiently physically active, most of the employees with more than two risk factors for NCD were inactive. Therefore, our findings are in agreement with previous research that reported habitual levels of physical activity were inversely associated with health care expenditure [30]. Our findings are further supported by Hill et al., who found that members of a health plan that were inactive, obese and overweight had significantly higher healthcare costs than those without these risk factors [31]. The average total healthcare expenditure was nearly double among the employees in the high-risk group than those who were low risk [31]. This difference in healthcare expenditure was higher than that reported in our study, and might be due to Hill and colleagues including adults over the age of 65 years in their study. In addition, healthcare expenditure in our research study was for a 12-month period, while Hill examined expenditure over a 17-month period. Therefore, difference in the number of doctors’ visits and healthcare costs might be greater between those with more than, versus those with less than 2 risk factors, if we had followed them for a longer period of time.

### Strengths and limitations

This research study has two key strengths. Firstly, the researchers were able to obtain healthcare expenditure data for those employees who did not attend the wellness days. We were, therefore, able to compare the healthcare expenditure of participants and non-participants. However, we were unable to determine their reasons for not participating. It is possible that some employees were unable to participate in the screening activities, as they were off-site or traveling when the wellness day took place. Secondly, most of the clinical measures were not self-reported, but measured by trained personnel, thereby verifying the health status of participants. Because the wellness days are predominantly a screening activity for large numbers of people, fasting blood glucose and serum triglyceride concentration were not measured. Despite this limitation, we were able to compare the clinical measures obtained in our study to findings from other similar research studies.

Self-reported physical activity could be viewed as a limitation for this research study, as objective measures might be able to provide more accurate data. However, self-reported measures of physical activity have been generally accepted among researchers due to their lower cost (than objective measures) and feasibility when including larger numbers of participants [32,33].

Healthcare expenditure data was available for all employees who were beneficiaries of the private health insurer. These data were limited to those expenses, which were claimed from the health insurer, and do not include out-of-pocket expenses. Since only approximately 16% of South Africans have private health insurance [19], these findings are unlikely to be generalizable to the non-insured individuals. Another limitation of the research study is that a large number of employees did not participate in the wellness days. As a result, selection bias can occur whereby the healthier and more motivated employees attend the wellness days. These employees might therefore have better health seeking behavior and lifestyles than those who do not attend. Despite these limitations, this research study provides some insight into the relationship between physical activity and increased risk for NCD. In addition, it is among the first research studies investigating the relationship between NCD risk and healthcare expenditure in South African employees.

## Conclusions

There are research studies that have examined the effect of a combination of lifestyle factors on cardio-metabolic risk in employed persons presenting for health risk appraisal [8,27]. However, there are fewer studies that have investigated whether individuals who meet physical activity guidelines also have other healthy lifestyle behaviors and reduced number of risk factors for NCD.

This research study has shown that more than two thirds of employees did not meet the recommended physical activity guideline of 150 minutes of moderate to vigorous intensity physical activity per week. These employees had a higher number of additional risk factors for NCDs compared to those who were sufficiently physically active. Moreover, those employees with two or more risk factors for NCD had significantly higher healthcare expenditure related to doctor’s visits than those with fewer risk factors.

These results suggest that employees are at increased risk for non-communicable diseases and would benefit from worksite health promotion programs. The interventions should include the promotion of habitual physical activity, as most employees were not meeting the guidelines. We anticipate that participation in the intervention programs has the potential to change physical activity behavior, improve the employees’ health status and play a role in reducing future healthcare expenditure. Additional research studies are required to determine the potential health and economic benefits of participation in physical activity-based worksite health promotion programs.

## Competing interests

TKA and EVL: There are no financial or other competing interests.

JC: At the time of writing the manuscript, JC was employed by Discovery Health Vitality, however, he was not involved in the data analysis.

## Authors’ contributions

TKA and EVL conceptualized the research study and performed data analysis and interpretation and writing manuscript. JC: Data acquisition and editing manuscript. All authors read and approved the final manuscript.

## Pre-publication history

The pre-publication history for this paper can be accessed here:

http://www.biomedcentral.com/1471-2458/13/1213/prepub
